# The effect of motorboat sound on Australian snapper *Pagrus auratus* inside and outside a marine reserve

**DOI:** 10.1002/ece3.4002

**Published:** 2018-06-13

**Authors:** Allen F. Mensinger, Rosalyn L. Putland, Craig A. Radford

**Affiliations:** ^1^ Biology Department University of Minnesota Duluth Duluth MN USA; ^2^ Leigh Marine Laboratory Institute of Marine Science University of Auckland Auckland New Zealand

**Keywords:** acoustics, baited underwater video, fish behavior, hearing, human‐generated sound

## Abstract

Human‐generated sound affects hearing, movement, and communication in both aquatic and terrestrial animals, but direct natural underwater behavioral observations are lacking. Baited underwater video (BUV) were deployed in near shore waters adjacent to Goat Island in the Cape Rodney–Okakari Point Marine Reserve (protected) or outside the reserve approximately four km south in Mathesons Bay (open), New Zealand to determine the natural behavior of Australian snapper *Pagrus auratus* exposed to motorboat sound. BUVs worked effectively at bringing fish into video range to assess the effects of sound. The snapper inhabiting the protected area showed no behavioral response to motorboat transits; however, fish in the open zones either scattered from the video frame or decreased feeding activity during boat presence. Our study suggests that motorboat sound, a common source of anthropogenic activity in the marine environment can affect fish behavior differently depending on the status of their habitat (protected versus open).

## 1 INTRODUCTION

Since the industrial revolution, human‐generated (anthropogenic) noise has changed the soundscape of many terrestrial and aquatic environments (McDonald, Hildebrand, & Wiggins, 2006; Watts et al., 2007). International legislation, such as the US National Environmental Policy Act and the European Commission Marine Strategy Framework Directive, recognizes the need to assess and manage the biological impacts of human‐generated noise (Slabbekoorn et al., 2010). Recent studies have demonstrated that human‐generated sound can detrimentally affect animal hearing, communication, movements, and foraging (Shannon et al., 2015; Slabbekoorn et al., 2010). However, it is difficult to translate these effects into meaningful predictions about individual fitness and population‐level consequences, (Morley, Jones, & Radford, 2014) because animals could possibly move away from sound sources, the disturbance may be transient and the animals could compensate to prevent long‐term impacts (Bejder et al., 2006). Therefore, it is imperative there are more experimental studies performed on organisms that can be tracked to investigate directly whether common sources of human‐generated sound disrupt behavior and/or reduce survival (Simpson et al., 2016).

In marine environments, sources of human‐generated sound include boat sonar, seismic profiling by oil and gas companies, and increased commercial boat traffic with larger and faster cargo ships. Furthermore, many coastal regions around the world, which contain environmentally sensitive reefs, seagrass meadows, and marshes, are experiencing large increases in populations, resulting in significant growth in maritime transportation, fishing, and recreation activities that include motorized watercraft (Davenport & Davenport, 2006). For example, there were 12.5 million registered motorboats in the United States in 2010 (NMMA 2011) and 0.25 million recreational motorboats centered on Auckland, New Zealand, which represents one boat for every six residents. Therefore, motorboats are a common and increasing source of human‐generated sound, with emerging evidence that this sound could affect communication, orientation, and territorial behavior in fish (Whitfield & Becker, 2014). Unlike industrial sources of sound such as seismic surveys and commercial shipping, it is relatively straightforward to design studies that use motorboats in controlled experiments to test impacts of sound on aquatic organisms (Boussard, 1981; Whitfield & Becker, 2014).

Marine protected areas or marine reserves are important refuge areas for many fish especially recreational and commercially important species (Agardy, 1994; Edgar et al., 2014; Mora et al., 2006). However, these areas do not necessarily provide acoustical refuge because sound can travel long distances underwater, and restrictions on fishing do not necessarily apply to other recreational activity including motorized watercraft. These locations are often synonymous with high tourism and associated diving industry, increasing sound through both boat traffic and diving activity (Radford, Jeffs, Tindle, Cole, & Montgomery, 2005). Additionally, the margins of protected areas are often heavily fished by both commercial and recreational fishers with the sound projected unabated into the reserves. Therefore, the aim of this study was to understand the behavior of Australian snapper, *Pagrus auratus*, within and outside a marine protected area in their natural environment to a real‐world situation of boat sound exposure.

## 2 MATERIAL AND METHODS

The two sites for all of the experiments were the protected Cape Rodney–Okakari Point Marine Reserve (CROPMR, a fully no‐take marine protected area) 36°16′110″S, 174°47′653″E and an open, fished site Mathesons Bay, Leigh New Zealand 36°18′380″S, 174°48′270″E, with all deployments within 500 m of listed coordinates. At both sites, two contrasting depths (5 m and 20 m) were used for the experiments.

The two sites were separated by only four km and had similar bottom topography and slope. Rocky reefs are interspersed in both areas with rocky and/or sandy bottoms with intermittent patches of kelp. Seabed sediment facies or composition at both locations were characterized as megarippled coarse sand and gravels (MRCSG) or continuous sand cover. The MRCSG consisted of 76% sand and 23% gravel with a textural description of poorly sorted, gravelly coarse sand, while the continuous sand cover was 99% sand and characterized as moderately well sorted fine sand (Hume, Oldman, & Black, 2000).

**Figure 1:**
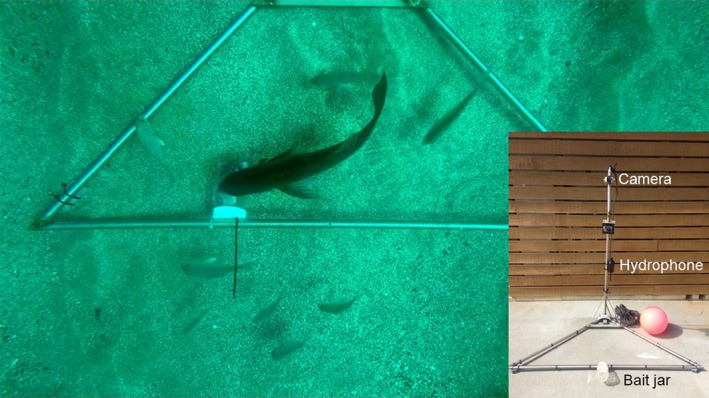
Baited underwater video (BUV) deployed in 5 m water in the marine reserve. Snapper are shown near the bait jar. Insert shows photograph of BUV. The triangular base measured 1.7 m × 1.2 m × 1.2 m

Baited underwater video (BUV) is a well‐used tool for determining fish densities in both marine reserves and fished sites (Willis & Babcock, 2000; Willis, Millar, & Babcock, 2000). The BUV array consisted of a triangular base of 22‐mm diameter stainless steel pipe measuring 1.7 × 1.2 × 1.2 m with a 1.2 m pole projecting upward from the intersection of the two shortest sides at approximately a 25° angle toward the center of the triangle. Lead weights were added to the base of the triangle to provide stability. A GoPro Hero 4 video camera was attached to the top of the pole facing the base. The camera was encased in the GoPro underwater housing modified with BacPac^™^ Backdoor Kit to allow the addition of a larger battery to extend recording time up to 4 hr. A SoundTrap 202 hydrophone was attached to the pole approximately 0.5 m from the base and set to record continuously at a sampling frequency of 144 kHz. A 2 L screw top clear plastic bottle was affixed with cable ties to the midpoint of the longest side of the base. Two to three 20 cm sl pilchard (*Sardinia neopilchardus*) were cross‐sectioned into approximately six equal sections and placed in the jar at the start of each trial for bait (Figure 1). Water temperature was taken from loggers (Hobo Water Temp Pro v2) at each site during deployment and ranged between 20.5 and 22.9°C at both sites.

A semi‐rigid 4.8 m boat equipped with a 4‐stroke 60 HP outboard motor was used for all trials. All BUV deployments were targeted to areas with minimal kelp to maximize fish observations. The BUV was deployed over the side of the boat in water depths of 5 m for shallow trials and 20 m for deep trials. Preliminary control experiments (*n* = 4) ranging from 30 to 60 min deployments without boat transit were conducted at both locations to determine the time needed for fish to reach steady numbers around the BUV and to also serve as controls for BUV deployment without boat presence. BUVs (*n* = 3) with unbaited bottles were also deployed at both Goat Island (*n* = 1) and Mathesons Bay (*N* = 2) to serve as controls to determine whether the apparatus attracted fish without bait.

Each BUV deployment consisted of a pre‐sound, sound and post‐sound period. The times for all boat activities were recorded and correlated with the internal clocks of the GoPro and hydrophone during post‐trial analysis to synchronize boat and fish activity. The pre‐sound interval consisted of a 20‐min submersion, which was not initiated until after deploying the BUV, moving the boat at least 200 m away from the site and turning off the engine (approximately 3 min after deployment). Following the pre‐sound interval, the engine was started and the boat made a direct path toward the BUV. The boat was sufficient distance away that startup sounds were not detectable by the recording devices. The sound phase was initiated once the sound of the boat was detected by the audio track of the GoPro camera (which was time synced to the hydrophone recording) and consisted of two consecutive passes made approximately 100 m past the BUV location from opposite directions. Following the second pass, the boat returned to the deployment site and made three consecutive 360^°^ circles around the BUV before departing the area and switching the engine off, which completed the sound phase. The duration of the sound phase was approximately 2.5 min. The post‐sound phase was initiated after the boat motor was turned off and was conducted for 15 min. After the post‐sound interval, the boat slowly approached buoy, the BUV was retrieved, and the bait exchanged prior to subsequent deployments.

Five deployments were conducted at each depth at each site. A minimum of four successful deployments with the BUV landing upright and the cameras not occluded by kelp were analyzed at each depth and each site over a 2‐month period. Adverse weather conditions (i.e., high winds) during this period often prevented small boat access to the sites resulting in a limited number of deployments. The total number of analyzed deployments were as follows: Mathesons Bay shallow (*n* = 4); Mathesons Bay deep (*n* = 5); Goat Island shallow (*n* = 4); and Goat Island deep (*n* = 4).

### 2.1 Behavior/video analysis

All video recording was conducted with the GoPro camera (fish lens option) with a recording rate of 29.97 frames per second and digitally stored on a 32 GB SD card. The viewing field of the camera was approximately 2.25 m^2^ which included most of the triangular base, the bait container and some area outside the triangle. The first 15 s of each minute of video recording following BUV deployment were examined during the pre‐ and post‐ sound phase while the entire sound phase was analyzed in consecutive 15‐s intervals. For statistical analysis, the last ten intervals of the pre‐sound phase and the first ten intervals of the post‐sound phase were compared with the 10 to 11 intervals that constituted the sound phase.

The video was examined for fish number, number of contacts with the bait container and number of intraspecific interactions between fish. As fish could enter and/or leave camera range window during each interval, fish number was determined to be the maximum number of fish in view at any time during the 15 s analysis period to avoid duplicate counting. During the fifteen‐second observation intervals in the absence of sound, it was rare for more than one fish to leave or enter camera range and the maximum counts were closely correlated with the number of fish observed during these periods. During the sound observation periods, it was more probable that more fish would exit the camera range than entering and therefore the maximum fish counts tended to be slightly higher than the number of fish remaining at the end of each interval. Bait container contacts were defined as the fish mouth contacting the bait jar or clearly discernable protrusions that attempted to bite the container. Consecutive attacks were treated individually if contact was lost and then re‐established with the bait container. Feeding frequency was calculated as the number of bites recorded per maximum number of fish observed per time interval.

### 2.2 Acoustic analysis

Hydrophone recordings from each deployment were manually inspected both aurally and visually using Audacity (version 2.0.6). Subsequent statistical analysis was performed using Matlab software (version R2014a) with codes specifically written for these recordings with the average SPLrms for ambient noise (pre‐ and post‐sound) and boat sound determined for each deployment. Power spectra of each phase of deployment were generated using fast Fourier transformation analysis with a Hanning window (1,024) and 50% overlap. Boat spectra were calculated using the highest power spectral density of the passage. This approach was used to give a standard integrative presentation of the sound across the frequency spectrum.

Particle acceleration generated by the motorboat transits were calculated in the center of the BUV frame using four hydrophones (HTI‐96‐MIN/3V/Low Noise; High Tech, Inc) recording simultaneously using a 4‐channel recorder (ST400; Ocean Instruments). These experiments were conducted without bait in the jar to decrease the possibility of variable fish density effecting the recordings. The hydrophones were arranged in a tetrahedral shape with a separation distance of 0.8 m. Therefore, the particle acceleration was calculated using Euler's equation of motion (Pierce, 1994):

(1) $$-\nabla p=\rho_{0}\frac{\partial u}{\partial t}$$

where ∇ is the gradient operator on the pressure $p,\;\rho_{0}$ is the fluid density, *u* is the velocity vector, and *t* is time. A discretized of Euler's equation in one dimension is:

(2) $$-\frac{P_{1}-P_{2}}{\rho_{0}d}=\frac{\partial u}{\partial t}$$

where, $P_{1}$ and $P_{2}$ are the sound pressure between two hydrophones separated by a distance *d*, and *u* is the velocity in line with the two points. Equation (2) lends itself to particle measurements because $P_{1}$ and $P_{2}$ can be measured with a hydrophone and it should be pointed out that the complex pressure should be measured and used. Using the centroid of the tetrahedral particle acceleration can be calculated in the *x*,* y*, and *z* directions using:

(3) $$a_{x}=\frac{P_{3}-\;P_{1}}{\rho_{0}d}$$

(4) $$a_{y}=\frac{2P_{2}-\left(P_{3}+\;P_{1}\right)}{\sqrt{3}\rho_{0}d}$$

(5) $$a_{z}=\frac{3P_{4}-P_{1}-\;P_{2}-\;P_{3}\;}{\sqrt{6}\rho_{0}d}$$

### 2.3 Statistical analysis

General linear models with negative binomial distributions were performed using the statistical software R (version 3.3.2) and factored site (protected verse fish); Depth (shallow verse deep) and intervals (pre‐sound, sound and post‐sound), as well as the interactions between each factor, against either the number of fish, number of bites, and frequency of bites. R packages used were “tidyr” (Wickham & Henry, 2017), “multcomp” (Hothorn, Bretz, & Westfall, 2008), “MASS” (Venables & Ripley, 2002), and “broom” (Robinson, 2017).

To ensure the GLMs suitably reflected the response variables a stepwise regression was performed removing each factor to find the best model. Six different models of the interactions between the three factors (site, depth, and interval) were performed for each response variable (frequency of bites, number of bites, and number of fish).

Sound pressure levels were compared using three‐way ANOVA, as the data met both homogeneity and normality assumptions. Root means squared (RMS) sound pressure levels (SPL) were reported as means ± 1 *SE*, where BUV video observations were reported as medians (1st quartile, 3rd quartile) as the data were nonparametric.

## 3 RESULTS

### 3.1 Control BUV drops

**Figure 2:**
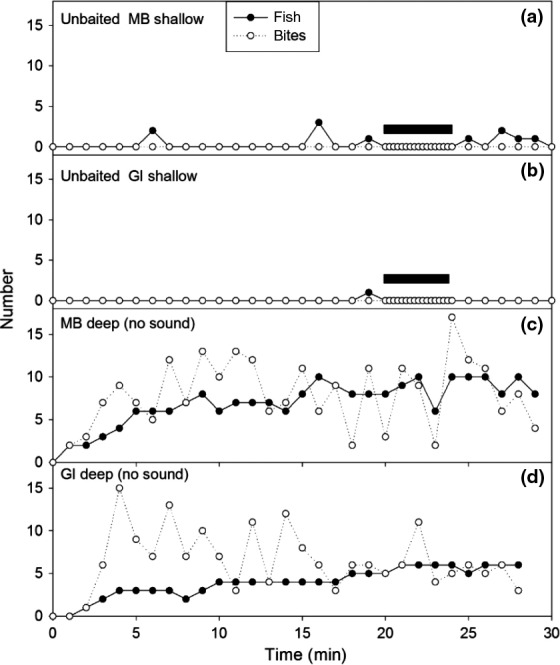
The number of fish (solid circles) at each site and bites (open circles) on the bait jar is plotted versus time after deployment. Each data point (fish number) represents the maximum number of fish visible in the video frame at any one time during a 15 s interval. Only the first 15 s of each observation minute in the pre‐, post‐sound, and no sound was quantified and plotted whereas all the 15‐s intervals were plotted during sound presentation. In a and b, the bait jar was empty. The solid rectangles indicate when the boat was transiting past the baited underwater video (BUV). In c and d, bait was placed in the jar, however, no boat transits were conducted during time interval. MB = Mathesons Bay (area open to fishing). GI = Goat Island (protected marine reserve). Shallow = 5 m; Deep = 20 m

Unbaited BUV were dropped at both Goat Island (*n* = 1) and Mathesons Bay (*n* = 2) to determine whether sufficient fish would frequent the area without bait attraction. Fish were rarely observed in the vicinity of the unbaited BUV and no interactions with the bait jar were observed (Figure 2a,b). BUV were then deployed at both sites with fish number peaking at approximately 20 min for both the protected and fished sites, which then was used as the minimum pre‐sound submersion time for the remainder of the study. Bite number was variable throughout the deployment in the absence of watercraft (Figure 2c,d).

**Figure 3:**
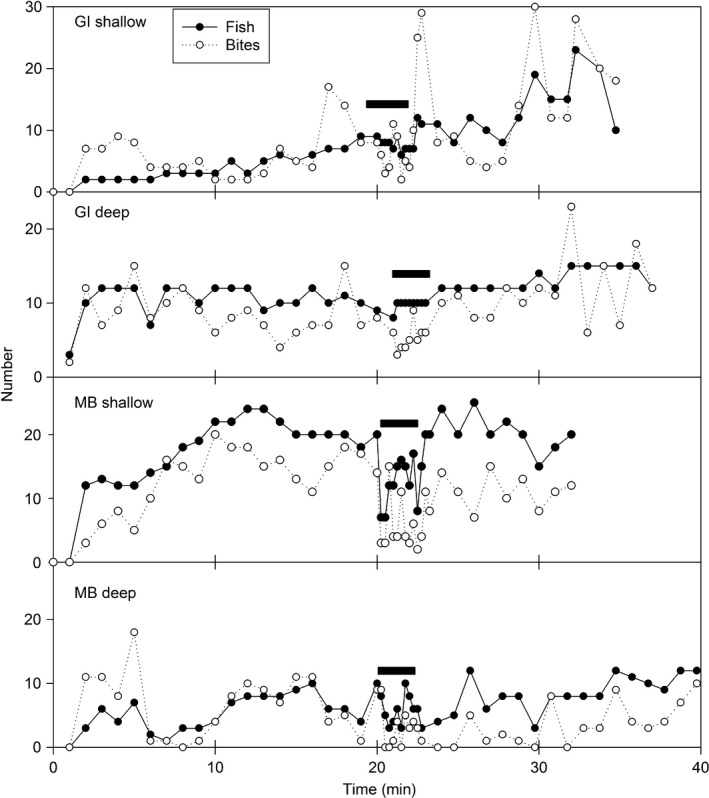
The number of fish (solid circles) and bites (open circles) is plotted versus time in minutes after deployment of the BUV for each site and depth. Each graph represents a single BUV deployment. The data point for fish number represents the maximum number of fish visible in the video frame at any one time during a 15 s interval. Only the first 15 s of each observation minute in the pre‐ and post‐sound period was quantified and plotted whereas all the 15‐s intervals were plotted during sound presentation. The boat transit through the BUV area is denoted by the solid rectangle. MB = Mathesons Bay (area open to fishing). GI = Goat Island (protected marine reserve). Shallow = 5 m; Deep = 20 m

Within minutes of deployment, fish were observed attacking the bait jar. There were several species attracted to the BUV, including John Dory (*Zeus faber*), leather jacket (*Parika scaber*), eagle ray (*Myliobatis tenuicaudatus*), spotted wrasse (*Notolabrus celidotus*), and red moki (*Cheilodactylus spectabilis*). However, at all sites, the visible area and contact with the bait jar were dominated by the Australian snapper (*Pagrus auratus*), therefore video analysis was restricted to this species for clarity of analysis. During boat transits, fish at the protected site were relatively indifferent to the boat, remained in view and continued attacking the bait jar (Figure 3a), while at the nonprotected site, both fish number and activity was reduced in the presence of the boat (Figure 3b).

### 3.2 Sound analysis

**Figure 4:**
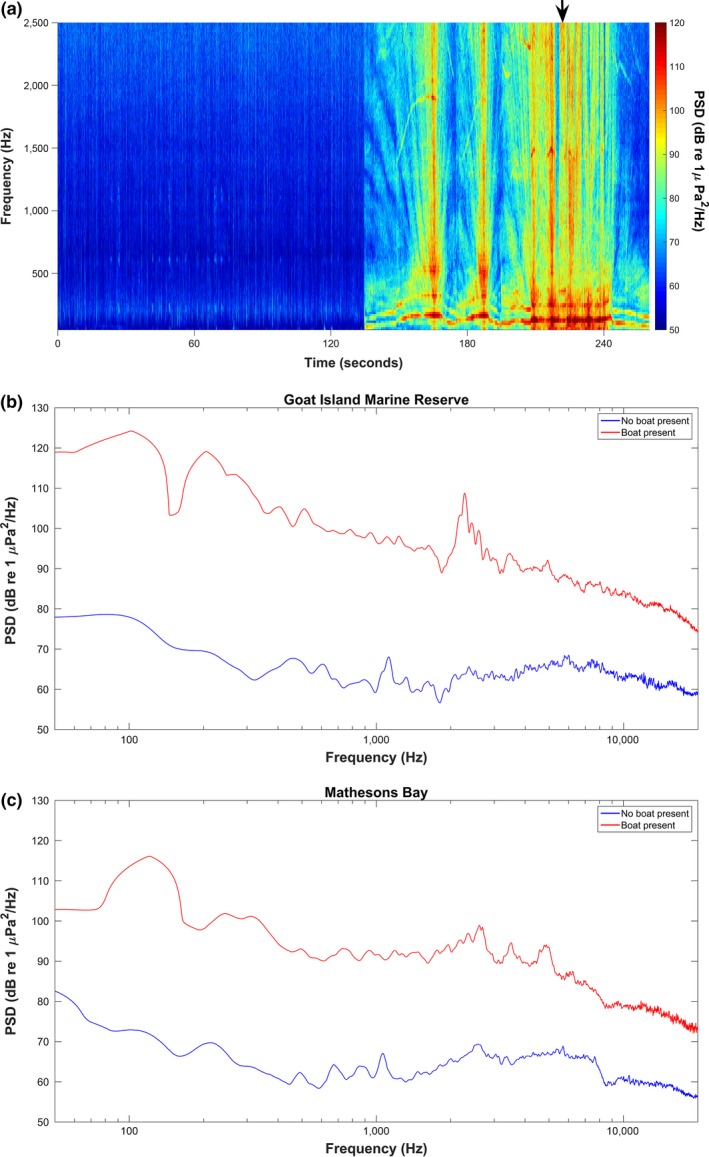
(a) Spectrogram of boat transit during one BUV shallow deployment at Mathesons Bay, color scale shows power spectral density. Peaks at 120 and 150 s represent boat transiting past BUV with sustained intensity (between 180 and 240 s on the *x*‐axis) representing boat circling the BUV; (b) example of shallow water (6 m) spectra from Goat Island; (c) example of deep water (20 m) spectra from Mathesons Bay. The blue lines represent ambient sound and the red lines represent ambient sound plus boat sound as taken from the highest power spectral density of the passage (example shown by arrow in Figure 4a)

**Figure 5:**
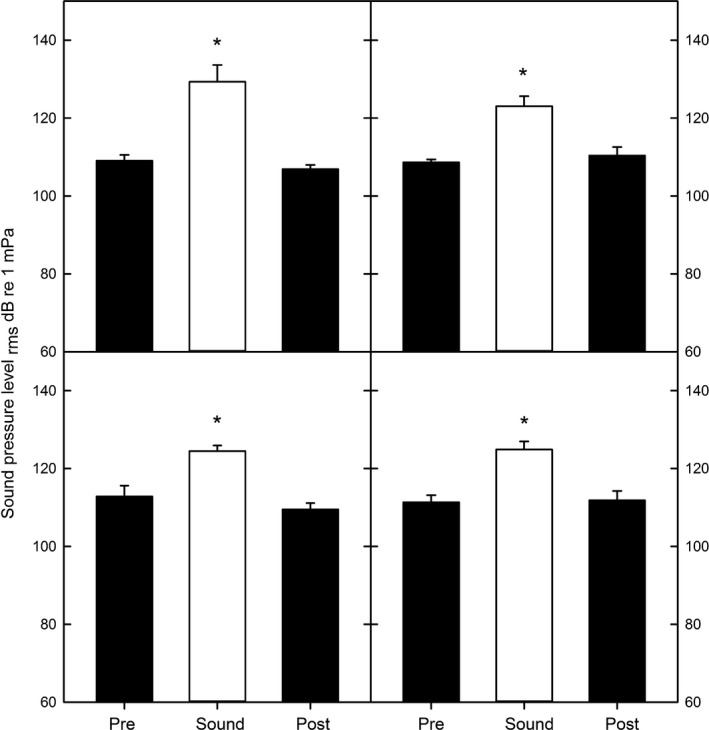
Bar graphs indicated the mean (+*SE*) SPL_rms_ in dB re 1 μPa at the four study sites during the pre‐sound (pre), sound and post‐sound (post) intervals. Asterisks indicated significantly different means

**Figure 6:**
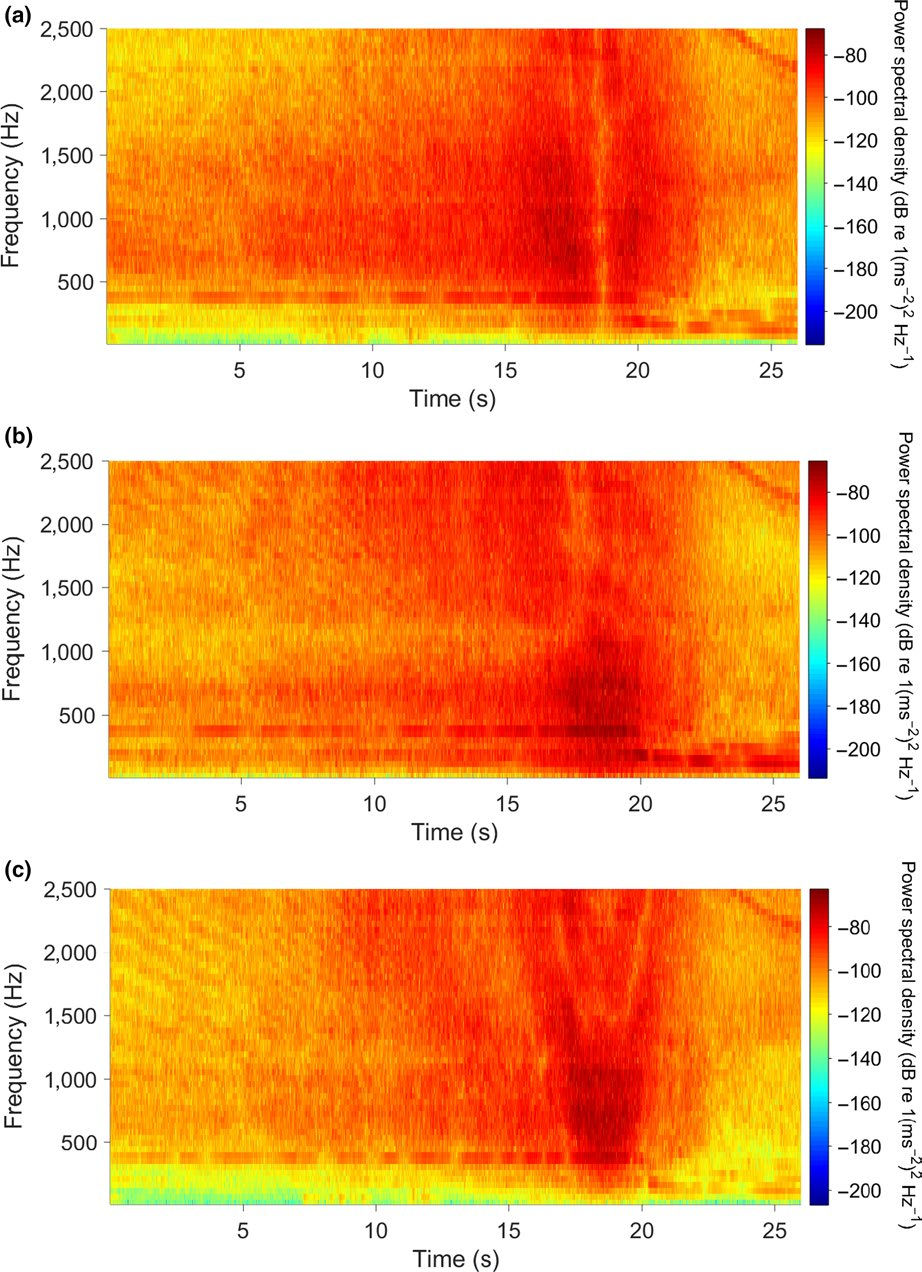
Particle acceleration spectrogram in the *x*‐ (a), *y*‐ (b), and *z*‐axis (c) of the boat transit during one BUV shallow deployment at Mathesons Bay, color scale shows power spectral density (dB re (1/ms^2^)^2^/Hz). Peak intensity was found between 100 and 1,100 Hz for all axes

Spectrogram analysis (Figure 4a) shows the three distinct passes or noisy periods created by the boat transiting and then circling the BUV stations. The motorboat sound also increased the sound levels across the entire frequency range (60 Hz–24 kHz), with the greatest increases observed below 2 kHz (Figure 4b,c). The ambient background levels between the two locations were also similar (Figure 4b,c – blue lines). The relatively quiet pre‐sound period is punctuated by two sharp increases in root‐mean‐square sound pressure level (SPL_rms_) that last approximately 10 s and span the entire frequency range (50–24,000 Hz) corresponding to the two transits past the BUV. The subsequent longer period (180 to 240 s) of sustained sound corresponds to the three 360° rotations around the BUV station. The overall ambient SPL_rms_ between the fished (111.3 ± 1.9 dB) and the protected (109.6 ± 1.7 dB) sites were similar (*F*_1, 69_ = 0.89, *p* = .34), while the increase in SPL_rms_ caused by the motorboat presence was also similar between the two sites (*F*_1, 69_ = 0.80, *p* = .86). At all four sites [protected deep (20 m) & shallow (5 m); fished deep (20 m) & shallow (5 m)], the boat presence caused a significant increase in SPL_rms_ of 15.95 ± 1.85 dB (*F*_1, 69_ = 60.27, *p* < .001) compared to the pre‐ and post‐sound intervals (Figure 5). Particle acceleration spectrogram analysis shows that there was energy in all three axes (*x*,* y*, and *z*) in the center of the BUV frame as the boat transited the area (Figure 6). The majority of the energy was found between 100 and 1100 Hz.

### 3.3 Fish numbers and behavior

The chosen model for statistical analysis of number of fish and number of bites was “Site × Intervals” which had a delta AIC > 2 from the next models, which were “Interval” and “Site” treated alone (Table 1). Furthermore, the *R* ^2^ values for the chosen models were 0.91 and 0.87, respectively, suggesting the clear majority of the variance was explained by site and intervals (Table 1). Similarly, the chosen model for statistical analysis of frequency of bites was also “Site × Intervals,” which had a delta AIC > 1.5 from the next models and an *R* ^2^ value of 0.75 (Table 1).

**Table 1:** Delta (Δ) AIC, weight, and *R* ^2^ for the six different general linear models performed in *R* for the three different response variables

	Frequency of bites			Number of bites			Number of fish		
	ΔAIC	Weight	*R* ^2^	ΔAIC	Weight	*R* ^2^	ΔAIC	Weight	i^2^
Site × Depth × Intervals	5.00	0.04	0.77	0.70	0.28	0.78	0.00	1.00	0.75
Site × Depth	0.20	0.40	0.75	31.10	0.16	0.85	47.70	0.00	0.84
Site × Intervals	10.40	0.00	0.75	37.00	0.00	0.87	92.10	0.00	0.91
Depth × Intervals	2.70	0.11	0.75	1.909	0.16	0.81	40.00	0.00	0.82
Site	9.00	0.00	0.74	31.10	0.00	0.85	73.80	0.00	0.89
Depth	0.00	0.43	0.74	1.90	0.00	0.81	46.20	0.00	0.83
Intervals	7.20	0.01	0.75	31.10	0.40	0.82	67.90	0.00	0.88

Row highlighted indicates the model used to generate statistical results on the three response variables.

**Figure 7:**
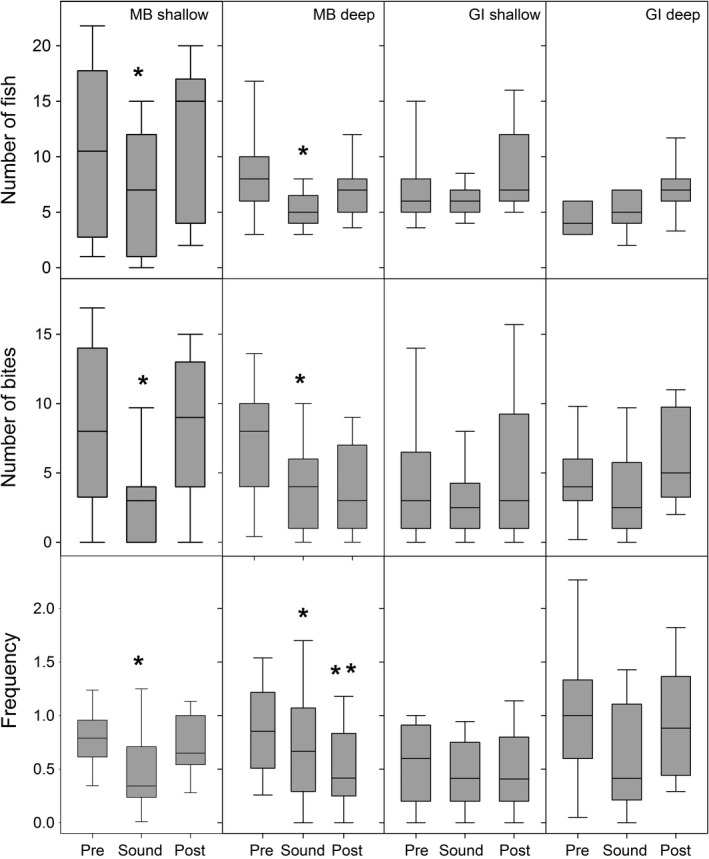
Each box shows the median value, the bottom and top of the box indicate 1st and 3rd quartiles, respectively, and the error bars indicate the 10th and 90th percentiles, for fish number (top), bite number (middle), and bite frequency (bottom) for pre‐sound, sound, and post‐sound activity. MB = Mathesons Bay; GI = Goat Island. The asterisks indicate statistically significant data (*p* < .05) was encountered following analysis with general linear models with negative binomial distributions

There was no significant difference in fish number observed at BUV stations within the protected site compared to the fished site (*z* value = 1.384, *p* = .1664). Interestingly, boat sound only influenced fish numbers at the fished site (site × sound interval; *z* value −4.146, *p* = .0001), where the number of fish significantly decreased during the motorboat transit compared to pre‐ and post‐sound intervals. However, this behavior was not observed at the protected site where fish numbers remained throughout deployment (Figure 7).

Bite number was similar between the fished and protected sites (*z* value = 0.891, *p* = .373). At the fished site during motorboat transit, the number of bites significantly (site × sound interval: *z* value = −4.016, *p* = .001),) decreased from pre‐ and post‐sound levels for both the shallow and deep sites. However, for the protected sites during motorboat transit, the number of bites remained constant throughout deployment (Figure 7). There was no significant difference in bite frequency between sites (*z* = −0.594, *p* = .55) or sound interval (*z* = 0.481, *p* = .63; Figure 7).

## 4 DISCUSSION

The present study demonstrates the direct impact of human‐generated sound on fish behavior. It also reveals that selective pressure in and outside a marine protected area can influence fish behavior in response to sound. For the first time, we show that snapper within a marine protected area respond differently to conspecifics found in fished areas when exposed to motorboat sound. This suggests that potential effects of human‐generated sound may be dependent on the protection status of their environment. For example, fish in the protected site are free from fishing and are known to exhibit bolder or more fearless behavior when confronted with recreational divers. However, fish outside the protected area are under both commercial and recreational fishing pressure and may have evolved or learned to be more cautious when detecting human intrusions (Cole, 1994; Smith & Anderson, 2016). This behavior is mirrored by reactions to motorboat sound where protected snapper remain in the area and continue feeding, whereas fish outside the protected area either leave the area or decrease feeding activity. This is consistent with earlier studies (Picciulin, Sebastianutto, Codarin, Farina, & Ferrero, 2010) that observed no changes to the behavior of the red‐mouthed goby (*Gobius cruentatus*) and the Mediterranean damselfish (*Chromis chromis*) exposed to motorboat sound in an Italian marine protected area.

The BUV's provided an effective mechanism for determining fish behavior in association with motorboat sound. The ambient sound levels were similar at the two sites indicating that the combination of environmental, propagation properties (similar bottom composition and topography) and human‐generated sound (boat traffic) was equal. Both sites received relatively light boat traffic during the weekday deployments with the research boat often the only motorized watercraft in the general vicinity which allowed the investigation to focus on a single source. The proximity of the two sites (4 km) allowed a direct comparison of the behavior independent of biotic (i.e., life history stage) and abiotic (i.e., temperature, bottom substrates) variables with the main difference being fishing pressure. The fished site is situated near the entrance of Leigh Harbour and weekend boat traffic and transits through the bay are common. The marine protected area receives approximately 300,000 visitors a year (http://www.doc.govt.nz), many of which participate in swimming, snorkeling, or scuba diving thus exposing the fish to a great number of human encounters.

There are several studies showing a range of effects of motorboat sound on marine animals (Holles, Simpson, Radford, Berten, & Lecchini, 2013; Nedelec et al., 2014; Simpson et al., 2016). For example, killer whales (Foote et al., 2006), humpback whales, (Risch, Corkeron, Ellison, & Van Parijs, 2012), and common dolphins (May‐Collado & Wartzok, 2008) have been shown to shift their call characteristics out of the frequency bands motorboat sound dominates as well as increasing sound levels (Foote, Osborne, & Hoelzel, 2004; Scheifele et al., 2005). Replayed motorboat sound disrupts the orientation behavior of larval reef fish (Holles et al., 2013), which is a critical stage in replenishing fish populations, and fish recruitment and larval survival was effected by motorboat sound, where Ambon damselfish (*Pomacentrus ambionenis*) exposed to motorboat sound had an increase in oxygen consumption and were more susceptible to predators (Simpson et al., 2016). Human‐generated sound has also been shown to affect different invertebrate species, with embryonic development of the sea hare (*Stylocheilus striatus*) effected by motorboat sound exposure (Nedelec et al., 2014). All these studies showed motorboat sound played a role in disrupting a key life history strategy, whether it be affecting larval development, reproduction success, predator avoidance, or communication signals. However, direct observation of the effect of sound on in situ fish behavior is complicated by the need to track the animals in environments with limited visibility and to ascertain that they are reacting to specific acoustic stimuli.

The fished site population reacted to sound by leaving the immediate area or altering their behavior. At both sites, fish would repeatedly attack the bait jar during the duration of the deployment despite limited reward. In the presence of the boat, this behavior in the nonprotected area dramatically declined as fish swam out of camera range or remained motionless. In the shallow portion of Mathesons Bay, the effect of the boat was transient as fish numbers and behavior returned quickly to pre transit levels, however, bite frequency remained depressed in the deeper water fish for at least 10 min after boat passage.

This study highlights the behavioral differences between fish inhabiting protected areas versus fished areas using BUV to monitor the fish in their natural environment when exposed to an environmental stressor, motorboat sound. The lack of fishing pressure could have selected for “boldness” within the snapper population inhabiting the protected area, which could expose them to greater sound intensity leading to reduced hearing sensitivity. In comparison, many of the nonprotected fish swam out of camera range as motorboat sound increased. However, the fish in the reserve showed little tendency to leave and may have been exposed to more sound. Whether this behavior is maladaptive and leads to reduction in hearing sensitivity remains to be determined. A previous study showed that fish exposed to ecotourism tend to have higher cortisol levels than fish that are protected from sound (Oliveira, Canário, & Bshary, 1999), which suggests these fish are stressed. Snapper (Caiger, Montgomery, & Radford, 2012) show a similar hearing ability to rainbow trout (Wysocki, Davidson, Smith, & Frankel, 2007), with greatest sensitivity to low‐frequency sounds (<400 Hz). Rainbow trout showed no effect of hearing loss after being exposed to sounds up to 150 dB re 1 μPa, therefore the exposure level of motorboat sound (125 dB re 1 μPa) in the present study is significantly less and unlikely to have caused any significant hearing damage to the protected fish. Also, snapper do not have ancillary hearing structures to promote greater sensitivity and bandwidth (Higgs & Radford 2013), hence are most sensitive to the particle motion component of the sound field (Radford et al., 2012). Motorboat particle motion measured in the center of the BUV frame suggests that the intensity is also not sufficient to cause any damage to the hearing of the fish. However, there is scarce research investigating the levels that particle motion fields need to be to effect fish hearing and this field requires more research.

An alternative hypothesis to the results is that the populations showed different behavior to the visual sighting of the boat. The waters in which the study was conducted were clear with sufficient light penetrating the water column that artificial lighting was not needed for video recording. However, the visual acuity of the adult snapper that dominated the BUV declines compared to juvenile estuarine fish, (Robinson, Jerrett, Black, & Davison, 2011) which would have lessened the distance that visual detection of the boat was possible. Additionally, on several occasions, the boat's shadow transited through the video recording frame with no startle response elicited by any of the fish, suggesting that visual cues were not precipitating behavior. During BUV retrieval, no behavioral changes were observed at either site suggesting visual targets did not influence the fish. In many instances, fish would follow the BUV during retrieval as it was pulled through the water column to the surface, indicating the fish were not influenced by the visual target presented by the boat or the motor at idling speed.

A range of stressors increasingly threatens reef ecosystems (Ferrari et al., 2015), yet reefs generate important revenue for many countries through tourism and provide food and livelihoods through fisheries. If sufficient resilience is to be retained for reef ecosystems to survive predicted global climate change, managing current, and local environmental stressors has been proposed as an essential goal. The present research highlights the need for human‐generated sound to be included in the design of marine protected areas, environmental management plans and, generally, the importance of assessing the direct consequences of human‐generated sound.

## CONFLICT OF INTEREST

None Declared.

## AUTHOR CONTRIBUTIONS

AFM and CAR designed the experiments, all authors contributed to data collection, video analysis was performed by AFM and sound analysis by RLP, and all authors contributed to manuscript writing.
